# Comparison of the free and bound phenolic profiles and cellular antioxidant activities of litchi pulp extracts from different solvents

**DOI:** 10.1186/1472-6882-14-9

**Published:** 2014-01-09

**Authors:** Dongxiao Su, Ruifen Zhang, Fangli Hou, Mingwei Zhang, Jinxin Guo, Fei Huang, Yuanyuan Deng, Zhencheng Wei

**Affiliations:** 1 Department of Food Science and Technology, Huazhong Agricultural University, Wuhan 430070, P.R. China; 2 Sericultural and Agri-food Research Institute, Guangdong Academy of Agricultural Sciences, Guangzhou 510610, P.R. China; 3 College of Food Science, Guangdong Pharmaceutical University, Zhongshan 528458, P.R. China

## Abstract

**Background:**

The phenolic contents and antioxidant activities of fruits could be underestimated if the bound phenolic compounds are not considered. In the present study, the extraction efficiencies of various solvents were investigated in terms of the total content of the free and bound phenolic compounds, as well as the phenolic profiles and antioxidant activities of the extracts.

**Methods:**

Five different solvent mixtures were used to extract the free phenolic compounds from litchi pulp. Alkaline and acidic hydrolysis methods were compared for the hydrolysis of bound phenolic compounds from litchi pulp residue. The phenolic compositions of the free and bound fractions from the litchi pulp were identified using HPLC-DAD. The antioxidant activities of the litchi pulp extracts were determined by oxygen radical absorbance capacity (ORAC) and cellular antioxidant activity (CAA) assays.

**Results:**

Of the solvents tested, aqueous acetone extracted the largest amount of total free phenolic compounds (210.7 mg GAE/100 g FW) from litchi pulp, followed sequentially by aqueous mixtures of methanol, ethanol and ethyl acetate, and water itself. The acid hydrolysis method released twice as many bound phenolic compounds as the alkaline hydrolysis method. Nine phenolic compounds were detected in the aqueous acetone extract. In contrast, not all of these compounds were found in the other four extracts. The classification and content of the bound phenolic compounds released by the acid hydrolysis method were higher than those achieved by the alkaline hydrolysis. The aqueous acetone extract showing the highest ORAC value (3406.9 μmol TE/100 g FW) for the free phenolic extracts. For the CAA method, however, the aqueous acetone and methanol extracts (56.7 and 55.1 μmol QE/100 g FW) showed the highest levels of activity of the five extracts tested. The ORAC and CAA values of the bound phenolic compounds obtained by acid hydrolysis were 2.6- and 1.9-fold higher than those obtained using the alkaline hydrolysis method.

**Conclusions:**

The free and bound phenolic contents and profiles and antioxidant activities of the extracts were found to be dependent on the extraction solvent used. Litchi exhibited good cellular antioxidant activity and could be a potentially useful natural source of antioxidants.

## Background

The regular consumption of fruits and vegetables has long been associated with the prevention of many chronic diseases, including cardiovascular disease and cancer, as evidenced by the results of clinical trials and epidemiological studies [1]. Oxidative stress is believed to be an important contributing factor in the development of these diseases, and the potent antioxidant properties of phytochemicals, including the phenolic compounds found in fruits and vegetables especially, may help to prevent the effects of oxidative stress on the body [2].

Litchi (*Litchi chinensis* Sonn.), which is a subtropical fruit, is fast becoming popular throughout the world because of its attractive appearance and delicious taste [3]. Although the phenolic constituents in the pericarp and seed of litchi have been well characterized [4-6], very few studies have been reported pertaining to the phenolic profiles of litchi pulp extracts in terms of their potential health benefits.

It is well known that phenolic compounds exist in both free and bound forms in plant cells, and that the free phenolic compounds are solvent extractable. In contrast, the bound phenolic compounds, which are covalently bound to the plant matrix, cannot be extracted into water or aqueous/organic solvents mixtures [7]. Although the total phenolic contents and antioxidant activities of litchi pulp have been reported previously [8-10], these studies only considered the solvent extractable free phenolic compounds present in the pulp. J Sun, YF Chu, Xz Wu and RH Liu [11] reported that about 4–57% of the phenolic compounds present in fruits existed in their bound forms. With this in mind, the phenolic contents and antioxidant activities of different fruits could well be underestimated, to a large extent, if the bound fractions are not considered in some way.

Furthermore, the extraction procedure used for the isolation of the phenolic compounds from plant materials could have a significant impact of the outcome of any investigation aimed at evaluating the properties of the phenolic compounds in plant materials, because it would dictate the nature and quantity of the phenolic compounds obtained in the extracts [12]. During their investigation of the extractive capability of various aqueous solvent systems (i.e., methanol, acetone and chloroform) A Ghasemzadeh, HZ Jaafar and A Rahmat [13] found that aqueous methanol afforded the highest level of phenolic and flavonoid compounds from the leaves, stems and rhizomes of two young ginger varieties. A higher phenolic extraction efficiency could be realized with an aqueous ethanol or methanol system, whereas the specific extraction of flavan-3-ols and proanthocyanidins could be achieved using acetone-based mixtures [14]. It is therefore important to select the most appropriate solvent for the extraction of phenolic compounds to allow for a high level of extraction efficiency. Furthermore, the bound phenolic compounds are covalently conjugated to cellulose, pectin and polysaccharides through ester bonds, and can be difficult to hydrolyze [15,16]. Alkaline, acidic or enzymatic hydrolysis methods can be used to release bound phenolic compound. In most of the studies conducted in this area, the bound phenolic compounds were released by alkaline hydrolysis, including, for example, our own research towards the preparation of litchi bound phenolic compounds [11,17-19]. In contrast, Bonoli et al. [14] reported the use of acid hydrolysis, and claimed that this method was more effective for the release of bound phenolic compounds than alkaline hydrolysis. In our previous study, we demonstrated that litchi pulp contained a high content of total phenolic and flavonoid compounds, although the procedure used for the extraction of the free and bound phenolic compounds in this particular study was based on a series of methods designed specifically for the extraction of other unrelated materials [19]. Thus, it still remains unclear as to what the most efficient solvent system would be for the extraction of free and bound phenolic compounds from litchi pulp.

Chemical antioxidant activity assays, such as ferric reducing/antioxidant power (FRAP) and oxygen radical absorbance capacity (ORAC), are commonly used in food studies, but these methods cannot account for biological activity. Biological systems are considerably more complex than simple chemical mixtures, and antioxidants may operate via multiple mechanisms [20]. The ability of chemical antioxidant activity assays to predict in vivo activity has been called into question on several occasions because none of these methods take account of the bioavailability, uptake and metabolism of the antioxidants. As a model based on cell culture, cellular antioxidant activity (CAA) represents a more biologically relevant method for the determination of antioxidant activity than the more commonly used “test tube” chemistry methods [2]. Using CAA to estimate the antioxidant activity of litchi pulp could therefore enhance our knowledge and understanding of the biological activity of litchi pulp.

The aim of the current investigation was to compare the differences in the extracts of litchi pulp, in terms of their total phenolic contents, phenolic profiles and antioxidant activities, when the free phenolic compounds had been extracted with different solvent systems and the bound phenolic compounds had been released using acid and alkaline hydrolysis methods. Using these results, it would then be possible to select appropriate methods for the extraction of free and bound phenolic compounds from litchi pulp.

## Methods

### Materials

The variety *Feizixiao*, which is one of the main litchi cultivars in South China, was purchased from a local fruit market in Guangzhou, China. The litchi fruit was carefully examined and identified by Professor Liangxi Ou, South China fruit germplasm resources evaluation scientist of Fruit Tree Research Institute, Guangdong Academy of Agricultural Sciences. A voucher specimen (NO 20120515) has been preserved in the refrigeration house (-20°C), Faculty of Horticulture, South China Agricultural University. Fully ripened fruits with brightly coloured red skins were selected, washed, peeled and de-seeded. Approximately 150 g of litchi pulp was used for each treatment.

### Chemicals

Gallic acid, chlorogenic acid, (+)-catechin hydrate, vanillic acid, caffeic acid, syringic acid, epicatechin, 4-methylcatechol, coumarin, ferulic acid, resveratrol, quercetin, 3,4-dihydroxybenzoic acid, *p*-coumaric acid, rutin, sinapic acid, 6-hydroxy-2,5,7,8-tetramethylchroman-2-carboxylic acid (Trolox), Folin–Ciocalteu’s phenol reagent, 2′,7′-dichlorofluorescin diacetate (DCFH-DA), fluorescein disodium salt, 2,2′-azobis (2-amidinopropane) dihydrochloride (ABAP) and quercetin dehydrate were purchased from Sigma-Aldrich (St. Louis, MO, USA). Dulbecco’s Modified Eagle’s Medium (DMEM), dimethyl sulphoxide, HPLC-grade acetic acid and acetonitrile were obtained from Thermo Fisher Scientific (Waltham, MA, USA). Human liver cancer cells (HepG2 cells) were obtained from the American Type Culture Collection (ATCC) (Rockville, MD, USA). Fetal bovine serum (FBS) was obtained from Haoyang Biologicals (Tianjin, China). Deionised water was prepared using a Milli-Q water purification system (Billerica, MA, USA).

### Extraction of free phenolic compounds from litchi pulp

The free phenolic compounds were extracted according to a published procedure [11]. Briefly, 150 g of freshly prepared litchi pulp was blended for 5 min in chilled solvent (80% methanol, 80% ethanol, 80% acetone, 80% ethyl acetate or deionised water; 1:2, w/v) using a Philips blender (Philips, Zhuhai, China). The samples were then homogenised using a XHF-D homogeniser (Ningbo Xin-zhi-Bio Technology Co. Ltd., Ningbo, China) at 5000 rpm for 5 min at 4°C. The homogenates were centrifuged at 5000 rpm for 8 min (Changsha Xiangzhi Instrument Co. Ltd., Changsha, China). The pellets were re-extracted with 300 mL of the original solvent and the supernatants were combined and evaporated to near dryness *in vacuo* on a rotary evaporator at 45°C. The concentrated supernatant was then reconstituted with methanol/water (85:15, v/v) and stored at -80°C. For the water extraction, the samples were extracted on a rocker incubator (Shanghai Yuejin Medical Instrument Factory, Shanghai, China) at room temperature for 120 min.

### Extraction of bound phenolic compounds from litchi pulp

The bound phenolic compounds were extracted from the residue resulting from the free phenolic extraction using 80% acetone. Six portions of the residue were prepared for the determination of the bound phenolic compounds in the litchi pulp.

### Alkaline hydrolysis method

The bound phenolic compounds in the litchi samples were extracted using a modified version of the alkaline hydrolysis method reported by M Bonoli, V Verardo, E Marconi and MF Caboni [14]. Briefly, the residue was digested in 2 M sodium hydroxide and stirred at room temperature for 18 h with shaking under nitrogen gas. The mixture was then neutralised with concentrated hydrochloric acid before being extracted six times with ethyl acetate. The organic fractions were combined and evaporated to dryness *in vacuo* at 35°C. The phenolic compounds were reconstituted in methanol/water (1 M; 85:15, v/v) and stored at -80°C.

### Acid hydrolysis method

The bound phenolic compounds in the litchi pulp were extracted with acid hydrolysis method according to a previously described method [21]. Briefly, 30 g samples of litchi residue were treated with 120 mL of methanol/H_2_SO_4_ (90:10, v/v) at 85°C for 20 h, and the resulting mixtures were neutralised with 10 M sodium hydroxide before being extracted six times with ethyl acetate. The organic fractions were combined and evaporated to dryness *in vacuo* at 35°C before being reconstituted with methanol/water (85:15, v/v) and stored at -80°C.

### Determination of the phenolic contents in litchi pulp

#### Total phenolic contents

The total phenolic contents were determined using the Folin–Ciocalteu (FC) colourimetric method described by Dewanto et al. [22]. Briefly, an aliquot (125 μL) of each of the extracts described above or a standard solution was mixed with 0.5 mL of deionised water and 125 μL of the FC reagent. After 6 min, 1.25 mL of a 7% Na_2_CO_3_ solution was added to the mixture, followed by 1.0 mL of water to bring the final volume to 3.0 mL. After 90 min of incubation at ambient temperature in the absence of light, the absorbance at 760 nm was measured using a Shimadzu UV-1800 spectrometer (Shimadzu Inc., Kyoto, Japan). Gallic acid was used as the standard, and the total phenolic contents were expressed as mg gallic acid equivalents (GAE)/100 g FW.

#### Total flavonoid contents

The total flavonoid contents were determined using the method reported by Dewanto et al. [22]. Briefly, an aliquot (250 μL) of each extract or a standard solution was mixed with 1.25 mL of deionised water followed by 75 μL of a 5% NaNO_2_ solution. After 6 min, 150 μL of a 10% AlCl_3_ · 6H_2_O solution was added to each mixture. After 5 min, 0.5 mL of 1 M NaOH was added, and the total volume was adjusted to 3.0 mL with deionised water. (+)-Catechin was used as a standard. The absorbance at 510 nm, which was corrected using a blank, was then determined and the results were expressed as mg of (+)-catechin equivalents (CE)/100 g FW.

#### Tannin contents

The tannin contents of the litchi pulp were determined using the method reported by Julkunen-Titto [23]. An aliquot (200 μL) of each extract or the standard solution was mixed with 3.0 mL of a 1:1 (v/v) mixture of 4% vanillin in methanol and 30% H_2_SO_4_ in methanol, and the resulting mixture was incubated for 20 min at room temperature in the absence of light. The absorbance at 510 nm was then read using a Shimadzu UV-1800 spectrometer. (+)-Catechin was used as a standard. The contents of the condensed tannin in the free fraction and the hydrolysed tannin in the bound fraction of the litchi pulp were expressed as mg of (+)-catechin equivalents (CE)/100 g FW.

### Analysis of phenolic compound compositions in litchi pulp

The phenolic compositions of the litchi pulp samples were determined using an HPLC-DAD method [19]. The HPLC separations were performed on an Agilent Zorbox SB-C_18_ column (250 × 4.6 mm, 5 μm, Palo Alto, CA, USA) at a flow rate of 1.0 mL/min using an injection volume of 20 μL. The binary gradient consisted of solution A (water/acetic acid 996:4 v/v) and solution B (acetonitrile). The gradient conditions were as follows: 0–40 min, solution B 5–25%; 40–45 min, solution B 25–35%; and 45–50 min, solution B 35–50%, followed by a 5 min equilibration period with 5% solution B. Each injection was monitored at 280 nm. The identity of each peak was confirmed based on the retention time determined for each pure compound. Values are expressed as μg/100 g FW.

### Assay of antioxidant capacity of litchi pulp

#### Determination of ORAC

The ORAC was performed according to the method described by Wolfe et al. [24]. The phenolic extracts were diluted with 75 mM phosphate buffer (pH = 7.4). The assay was performed in black-walled 96-well plates (Corning Scientific, Corning, NY, USA). Each well contained 20 μL of the extract or 20 μL of the trolox standard (range, 6.25–50 μM) or a blank and 200 μL of fluorescein (final concentration of 0.96 μM). The plates were incubated at 37°C for 20 min on an Infinite M200pro plate reader (Tecan Austria GmbH, Salzburg, Austria). After incubation, 20 μL of 119 mM ABAP, which was freshly prepared for each run, was added to each well except the F well, which was treated with 20 μL of 75 mM phosphate buffer. The fluorescence conditions were as follows: excitation at 485 nm and emission at 520 nm for 35 cycles at intervals of 4.5 min. The ORAC results were expressed as μmol trolox equivalents (TE)/100 g FW.

#### Measurement of CAA

The CAA assay was conducted according to the method described by Wolfe et al. [24]. Briefly, HepG2 cells were seeded at a density of 6 × 10^4^/well in a black 96-well microplate in 100 μL of growth medium (DMEM medium containing 10% fetal bovine serum). The growth medium was removed after 24 h, and the triplicate wells were then treated for 1 h with 100 μL portions of different concentration of quercetin or the litchi pulp extracts plus 25 μM DCFH-DA in DMEM. The cells were then treated with 100 μL of 600 μM ABAP in PBS, and the fluorescence was measured with an Infinite M200pro plate reader using an excitation wavelength of 485 nm and an emission wavelength of 520 nm for 12 cycles at 5 min intervals. The CAA values were calculated according to the method of Wolfe et al. [24]. The CAA results are reported as micromoles of quercetin equivalents (QE) per 100 g of lychee pulp FW.

### Statistical analyses

All of the results are reported as the mean ± SD for triplicate determinations of each sample. The results of the acid and alkaline hydrolyses were analysed using the *t* test. A one-way ANOVA test was used, followed by an SNK-q test, to compare the extracts obtained from the different solvents. A value of *p* < 0.05 was considered statistically significant. All of the statistical analyses were performed using SPSS statistical package version 13.0 software (SPSS Inc. Chicago, IL, USA).

## Results

### Effects of the different solvent extraction on the content of free and bound phenolic compounds in litchi pulp

The contents of the free and bound phenolic, flavonoid and tannin compounds in the litchi pulp are shown in Tables 1 and 2. Aqueous acetone gave the highest yield for the extraction of the free phenolic, flavonoid and condensed tannin compounds, followed by aqueous mixtures of methanol, ethanol and ethyl acetate (*p* < 0.05). The water extract afforded the lowest contents for the different classes of phenolic compounds. The flavonoids content in the 80% aqueous methanol extract was not significantly different from that of the aqueous ethanol extract.

**Table 1:** Contents of the free phenolic, flavonoid and tannin compounds of the litchi pulp in various solvents extracts

	**Free phenolic compounds**				
	**80% methanol**	**80% ethanol**	**80% acetone**	**80% ethyl acetate**	**Water**
Phenolic (mg GAE/100 g FW)	190.69 ± 3.69b^1^	171.81 ± 3.42c	210.67 ± 9.85a	151.79 ± 5.70d	121.76 ± 4.23e
Flavonoid (mg CE/100 g FW)	85.55 ± 2.34b	80.84 ± 2.98b	103.88 ± 1.82a	65.17 ± 1.7c	57.15 ± 1.31d
Tannin (mg CE/100 g FW)	114.32 ± 3.33b	107.02 ± 1.45c	156.08 ± 2.85a	80.98 ± 2.69d	70.93 ± 1.98e

^1^Values not sharing a common letter in each row are significantly different (*p* < 0.05).

**Table 2:** Contents of the bound phenolic, flavonoid and tannin compounds of the litchi pulp in various hydrolysis extracts

	**Bound phenolic compounds**	
	**Acid hydrolysis**	**Alkaline hydrolysis**
Phenolic (mg GAE/100 g FW)	61.27 ± 1.78**^1^	30.71 ± 0.51
Flavonoid (mg CE/100 g FW)	29.74 ± 1.44**	18.16 ± 0.83
Tannin (mg CE/100 g FW)	37.37 ± 3.49**	15.86 ± 1.70

^1^Asterisk (**) within the same row indicate significant difference (*p* < 0.01).

For the bound phenolic compounds, the results revealed that the acid hydrolysis method led to the release of far more bound phenolic compounds from the residue of litchi pulp than the alkaline hydrolysis, where free phenolic compounds had been extracted with 80% acetone (*p* < 0.05). The contents of the phenolic, flavonoid and hydrolysed tannin compounds in the extract from the acid hydrolysis were determined to be 2.0, 1.6, and 2.3-fold greater than those in alkaline hydrolysis extract, respectively.

### Effects of different solvents on the composition of free and bound phenolic compounds in litchi pulp

Analysis by HPLC-DAD revealed that ten phenolic compounds were present in the litchi pulp extracts, including 3,4-dihydroxybenzoic acid, (+)-catechin, vanillic acid, caffeic acid, syringic acid, (-)-epicatechin, 4-methylcatechol, ferulic acid, rutin and quercetin (Tables 3 and 4). The number and type of phenolic compounds detected in the litchi pulp extracts obtained from different extraction solvents varied considerable (Table 3). All the phenolic compounds mentioned above were detected in the free forms from the litchi pulp extract obtained using 80% acetone except quercetin, which was not detected in the free form in any of the five solvent systems. In contrast, only seven of the ten phenolic compounds described above were detected in the extracts obtained from the other four solvents systems. 3,4-Dihydroxybenzoic acid was not extracted in any of these four solvent systems and syringic acid was not extracted when 80% methanol, 80% ethyl acetate or water was used as the extraction solvent. Although similar contents of syringic acid were detected in the litchi pulp extracts obtained when 80% acetone and 80% ethanol were used as the extraction solvents, vanillic acid was not detected in the latter of these two systems. Besides, the five extraction solvents exhibited significantly different extraction efficiencies for the nine compounds. As shown in Table 3, 4-methylcatechol, rutin and (-)-epicatechin were determined to be the major phenolic compounds extracted in their free form the litchi pulp in all five solvent systems. The highest 4-methylcatechol content was found in extracts obtained using the 80% acetone and 80% methanol solvent systems, followed by 80% ethanol, 80% Ethyl acetate and water. The latter two had similar extraction efficiencies for 4-methylcatechol (*p* > 0.05). Aqueous acetone afforded a 4-methylcatechol yield that was almost 2-fold greater than that of water (*p* < 0.05). The second most abundant phenolic compound extracted from the litchi pulp was rutin. The ranking order for the rutin yields given by the five different solvent systems was similar to that of 4-methylcatechol. The extraction efficiency of aqueous acetone for (-)-epicatechin was significantly higher than that of methanol, follow sequentially by 80% ethanol, 80% Ethyl acetate and water (*p* < 0.05). The use of 80% acetone allowed for the extraction of 0.5-, 1.1-, 1.5- and 7.7-fold more (-)-epicatechin than 80% methanol, 80% ethanol, 80% ethyl acetate and water, respectively. The concentrations of the other phenolic components were less than 500 μg/100 g FW. The orders of the extraction efficiencies for other phenolic compounds were similar except for water, which had the highest extraction efficiency for vanillic acid of all of the solvent systems, followed sequentially by acetone, methanol and ethyl acetate. As mentioned above, the 80% ethanol solvent system did not extract vanillic acid from litchi pulp.

**Table 3:** Free phenolic profiles of litchi pulp

**Phenolic**	**Free phenolic compounds**				
	**80% methanol**	**80% ethanol**	**80% acetone**	**80% ethyl acetate**	**Water**
3,4-dihydroxybenzoic acid	ND^1^	ND	115.26 ± 11.17^2^	ND	ND
(+)-catechin	140.34 ± 12.01ab^3^	131.66 ± 2.74bc	163.34 ± 21.13a	83.34 ± 5.27d	107.78 ± 25.53 cd
Vanillic acid	130.77 ± 19.50b	ND	126.48 ± 7.27b	72.99 ± 4.90c	254.25 ± 32.88a
Caffeic acid	393.37 ± 21.08b	188.95 ± 16.28d	442.93 ± 17.78a	244.68 ± 11.43c	38.66 ± 4.57e
Syringic acid	ND	30.55 ± 1.48	39.50 ± 6.17	ND	ND
(-)-epicatechin	1489.88 ± 159.63b	1118.81 ± 20.54c	2308.19 ± 106.54a	929.13 ± 18.89d	266.28 ± 13.99e
4-methylcatechol	5926.88 ± 475.53a	4938.91 ± 483.11b	6337.70 ± 209.91a	3493.87 ± 236.51c	3304.28 ± 148.94c
Ferulic acid	442.64 ± 19.31a	276.60 ± 21.05b	488.34 ± 68.82a	286.17 ± 86.28b	215.51 ± 13.89b
Rutin	3441.47 ± 212.65ab	3125.16 ± 94.31b	3580.33 ± 274.25a	2016.82 ± 108.41c	1614.32 ± 120.93d

^1^ND – not detected.

^2^Values expressed as μg/100 g FW.

^3^Values not sharing a common letter within the same row are significantly different (*p* < 0.05).

**Table 4:** Bound phenolic profiles of litchi pulp

**Phenolic**	**Bound phenolic compounds**^**b**^	
	**Alkaline hydrolysis**	**Acid hydrolysis**
(+)-catechin	14.02 ± 3.99^1^	ND^2^
Vanillic acid	ND	37.10 ± 4.88
Caffeic acid	ND	95.52 ± 2.44
Syringic acid	ND	4.98 ± 1.36
(-)-epicatechin	207.95 ± 23.70**^3^	50.57 ± 6.32
4-methylcatechol	ND	2404.97 ± 216.36
Rutin	83.11 ± 10.99**	13.08 ±0.96
Quercetin	28.77 ± 10.38	56.12 ± 7.73*

^1^Values expressed as μg/100 g FW.

^2^ND – not detected.

^3^Double asterisk (**) within the same row indicate significant difference (*p* < 0.01), asterisk (*) within the same row indicate significant difference (*p* < 0.05).

The types and quantities of the bound phenolic compounds released from the litchi pulp were influenced by the hydrolysis method (Table 4). Seven phenolic components were found in the extract following the acid hydrolysis of the litchi pulp residue, whereas only four phenolic compounds were found in the extract following alkaline hydrolysis. (-)-Epicatechin, rutin and quercetin were released by both the acid and alkaline hydrolyses. Four phenolic compounds, including vanillic acid, caffeic acid, syringic acid and 4-methylcatechol, were detected exclusively in the extracts resulting from the acid hydrolysis, whereas (+)-catechin was only found in extracts following the alkaline hydrolysis. The quantitative analysis of the extracts following the acid and alkaline hydrolysis treatments showed that 4-methylcatechol and (-)-epicatechin were the main phenolic components detected in the bound fractions of litchi pulp when the residues were extracted by acid or alkaline hydrolysis, respectively.

### Antioxidant capacity of litchi pulp extracts from different solvents extraction

**Figure 1:**
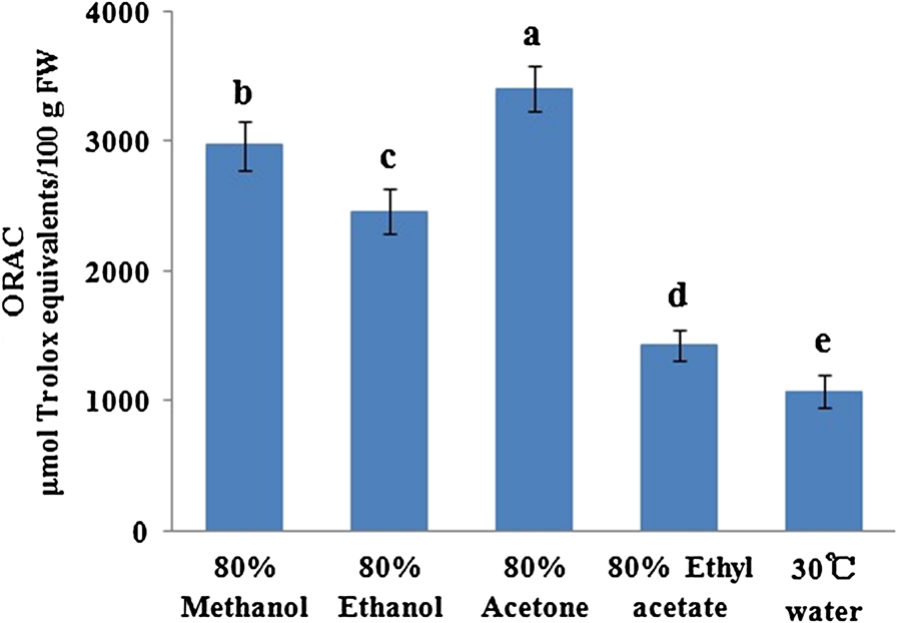
**ORAC values of the different solvent extracts of free phenolic compounds from litchi pulp.** Bars not sharing a common letter are significantly different (*p* < 0.05).

**Figure 2:**
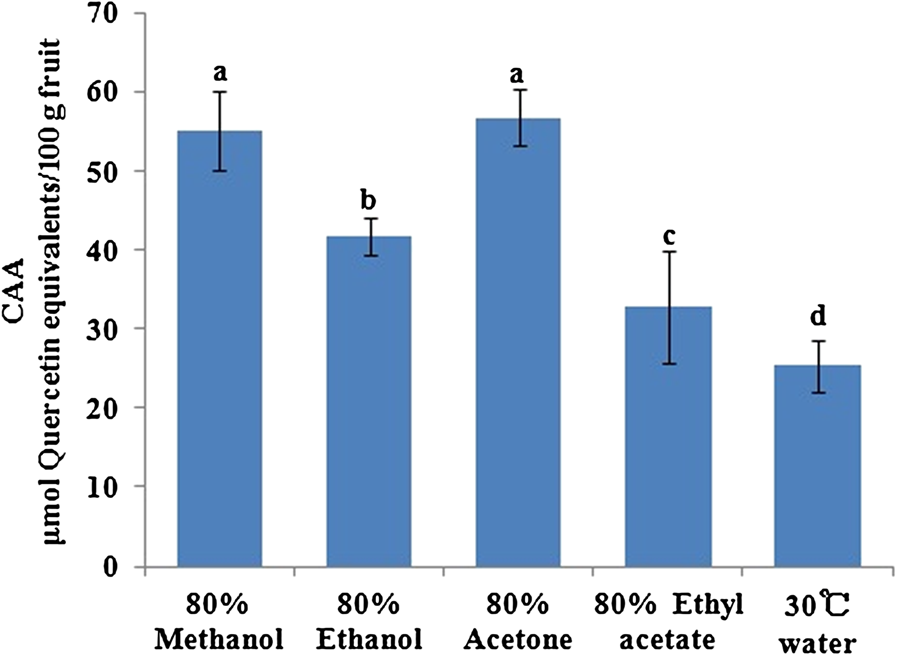
**CAA values of the different solvent extracts of free phenolic compounds from litchi pulp.** Bars not sharing a common letter are significantly different (*p* < 0.05).

The antioxidant activities of the litchi pulp extracts were evaluated using the ORAC and CAA assays. The antioxidant activity determined by ORAC for the free fraction is shown in Figure 1. The extracts obtained from the five different solvent systems showed significantly different ORAC values (*p* < 0.05) in the range of 1075.66 ± 129.88 to 3406.95 ± 176.14 μmol TE/100 g FW. The aqueous acetone extract had the highest antioxidant activity, followed sequentially by the methanol (2971.96 ± 187.01 μmol TE/100 g FW), ethanol (2461.09 ± 178.35 μmol TE/100 g FW) and ethyl acetate extracts (1433.08 ± 123.54 μmol TE/100 g FW). The lowest ORAC antioxidant activity was detected in the water extract. The ORAC value of the 80% acetone extract was higher than that of the 80% methanol extract by approximately 15% and was more than 2-fold higher than that of the water extract. The CAA values (Figure 2) of the acetone and methanol extracts, however, were equally the greatest (56.73 ± 3.53 and 55.13 ± 5.03 μmol QE/100 g FW, respectively) (*p* > 0.05). The activities of the solvent systems were of the same order as those observed in the ORAC assay, ranking as ethanol (41.77 ± 2.29 μmol QE/100 g FW) > ethyl acetate (32.78 ± 7.11 μmol QE/100 g FW) > water (25.35 ± 3.22 μmol QE/100 g FW).

**Figure 3:**
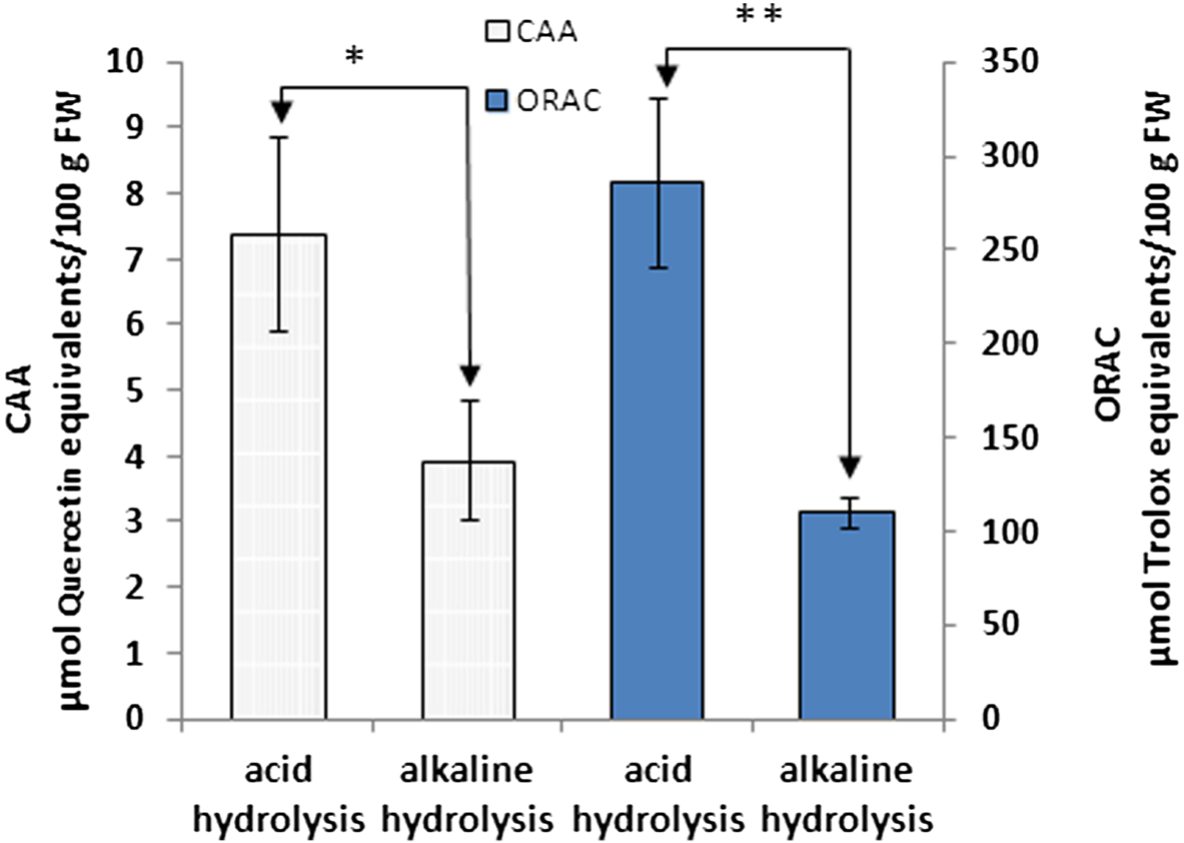
**CAA and ORAC values of the different hydrolysis extracts of bound phenolic compounds from litchi pulp.** Bars with an asterisk (*) are significantly different (*p* < 0.05), bars with a double asterisk (**) are significantly different (*p* < 0.01).

The CAA and ORAC values (7.40 ± 1.49 μmol QE/100 g FW and 286.14 ± 45.63 μmol TE/100 g FW) (Figure 3) for the bound phenolic compounds released by acid hydrolysis were 1.9 and 2.6-fold higher than those obtained by alkaline hydrolysis (3.95 ± 0.89 μmol QE/100 g FW and 110.16 ± 8.43 μmol TE/100 g FW) (*p* < 0.05).

## Discussion

### Effects of extraction solvents on the contents of free and bound phenolic compounds in litchi pulp extracts

The choice of solvent for the extraction of the polyphenolic compounds from plant materials is greatly important because different solvents influence the extraction efficiencies of the different phenolic components in different ways. The results of the current study revealed that 80% aqueous acetone extracted higher levels of phenolic, flavonoid and condensed tannin compounds from litchi pulp than any of the other solvent systems tested. These results were similar to those reported for litchi flowers by Liu et al. [25], where the acetone extracts of litchi flowers contained the highest total amounts of phenolic, flavonoid and condensed tannin compounds of the three solvent systems tested (i.e., acetone, methanol and water). Prasad et al. [4], however, reported that 50% aqueous ethanol extracts of litchi seeds contained the highest number of total phenolic compounds and showed the highest total antioxidant capacity, following their evaluation of five different polar solvents, including ethanol, 50% ethanol, methanol, 50% methanol and water. The phenolic extraction efficiencies of the different parts of litchi may be dependent on the polarities of the compounds that are being extracted and different solvents may therefore be required depending on the compounds contained in the different parts of litchi. Previous studies have shown that the use of an acetone-water mixture affords much higher yields of total phenolic compounds than that of a methanol–water mixture because these extracts contain more flavan-3-ols and conjugated forms of ellagic acid [12,26]. Aqueous acetone extracts generally contain more tannins than any other extract, and similar results were observed in the current study, where the 80% aqueous acetone extract contained much more condensed tannin than any of the other extracts. Furthermore, acetone displayed higher extraction efficiency for phenolic and flavonoid compounds than any of the other solvent systems used in the current study. Thus, the acetone mixture represents a more efficient solvent system for the extraction of free phenolic compounds from litchi pulp. The free phenolic content observed in the litchi pulp in the current study was much higher than those detected in the litchi cultivars from northern Mauritius [10] and southern Florida [27], where the phenolic compounds were extracted with acetone/water (70:30 v/v) and methanol (100%), respectively. As well as the different litchi genotypes tested, the observed discrepancies in the phenolic contents of these litchi pulps can also be attributed to differences in the solvent systems used for their extraction.

As mentioned above, there are some bound phenolic compounds in plants that cannot be extracted through solvent extraction. Enzymatic, thermal and alkaline hydrolysis methods have all been used to extract bound phenolic compounds from different sources, with alkaline hydrolysis being the most commonly used of these methods. In the current study, however, more bound phenolic, flavonoid and hydrolysed tannin compounds were obtained by acid hydrolysis than alkaline hydrolysis. As previously reported, far more bound phenolic compounds were released from apple and peach (80.3 and 52.8 mg GAE/100 g FW) by acid hydrolysis [28] than (4.9 and 3.2 mg GAE/100 g FW) alkaline hydrolysis [11], because the higher temperature used for the acid hydrolysis method allowed for the release of the bound phenolic compounds trapped in the cores or conjugated to cell wall dietary fibres or proteins [14,28]. The acid hydrolysis method would therefore be more suitable than the alkaline hydrolysis method for the extraction of bound phenolic compounds from litchi pulp.

### Effects of different extraction solvents on the compositions and contents of phenolic compounds in litchi pulp

The phenolic compound compositions in the free fraction were affected by the nature of the extraction solvent system. All nine compounds were found in the 80% acetone extract, whereas 3,4-dihydroxybenzoic acid and syringic acid were not detected in the 80% methanol, 80% ethyl acetate or water extracts. Furthermore, 3, 4-dihydroxybenzoic acid and vanillic acid were not detected in the 80% ethanol extract. It is noteworthy that the total contents of these three acids were less than 500 μg/100 g FW and therefore had little influence to the total phenolic content of the litchi pulp. The 4-methylcatechol, rutin and (-)-epicatechin levels, however, varied considerably depending on the solvent system used for the extraction, with the use of 80% acetone providing the highest levels of these compounds. M Kajdzanoska, J Petreska and M Stefova [12] compared a variety of different solvent mixtures for the extraction of phenolic compounds from strawberries and reported that the acetone extract contained all of the phenolic compounds that could be detected. Gallic acid, procyanidin B2 (-)-gallocatechin, (-)-epicatechin, and (-)-epicatechin-3-gallate have all been found in the pericarp [4,29] and seeds [5] of litchi, although (-)-epicatechin, which serves as one of the major procyanidin compounds in litchi seeds and pericarp, was found at much higher levels [4,5,29]. In our previous study, we showed that rutin and (-)-epicatechin were the main free phenolic compounds in litchi pulp [19]. It is noteworthy that similar phenolic compounds exist in different parts of litchi. The most abundant phenolic compound, 4-methylcatechol, which has not been previously reported in litchi pulp, was found in the current study, and this compound can lead to elevated levels of endogenous nerve growth factor in *vivo*[30]. As mentioned above, the acetone extracts of litchi pulp contained higher total amounts of phenolic compounds than those of the methanol solvent system. Similar levels of 4-methylcatechol and rutin, however, were found in the aqueous acetone and methanol extracts, indicating that these solvent systems performed equally as well for the extraction of these compounds. Furthermore, the condensed tannins in the extracts were detected using a chemical method, indicating that proanthocyanidins may present in litchi pulp. Arranz et al. [28] reported the presence of extractable proanthocyanidins in the methanol/acetone extracts of apple, nectarine and peach pulp by normal-phase HPLC [28].

As shown by our previous research, four hydrolysable compounds were released from the litchi pulp residue, although seven individual phenolic compounds, including compounds generally associated with the alkaline hydrolysis of bound phenolic compounds, were detected in extracts obtained from the acid hydrolysis preparation. Ferulic acid, *p*-hydroxybenzoic acid, catechin, gallocatechin, gallic acid, and caffeic acid were identified as the main bound phenolic compounds released by the acidic hydrolysis of 26 different types of fruit that are regularly consumed as part of a typical Spanish diet [31]. Caffeic acid was identified in the litchi pulp in the current study at similar levels to those reported in the literature. The major bound phenolic compounds isolated from litchi were (-)-epicatechin and 4-methylcatechol, which could be attributed to different fruits being tested. Acidic hydrolysis represents an effective method for the release of bound phenolic compounds trapped in the cores or the fruits and bound to cell wall matrix [28]. The use of the acid hydrolysis method therefore allowed for the detection of higher levels of bound phenolic compounds and hydrolysable tannins. It is noteworthy, however, that some hydroxycinnamic acids may be lost during the acidic hydrolysis of the residues.

The phenolic compounds discussed in the current study were identified based only on their retention time by HPLC analysis and the use of other analytical methods such as HPLC-MS and NMR would be necessary to allow for the structures of these compounds to be determined beyond a reasonable doubt.

### Antioxidant activity of free and bound phenolic compounds of litchi pulp

Litchi pulp contains a high level of phenolic compounds and in our previous study we found that both the free and bound phenolic compounds from litchi pulp exhibited excellent antioxidant activities based using DPPH and FRAP assays [19]. In the current study, the aqueous acetone extracts exhibited higher ORAC values than the methanol extracts, most likely because higher levels of (-)-epicatechin were found in the acetone extracts. The (-)-epicatechin exhibited good antioxidant activity based on the results of the ORAC assay [32]. The 80% acetone extracts therefore exhibited higher ORAC values than the 80% methanol extracts.

The CAA values for the different extracts, however, appeared in a different order to those obtained from the ORAC assay. The acetone and methanol solvent extracts had similar CAA values, which were higher than those of the other extracts. Of the three phenolic compounds, rutin exhibited no activity in the CAA assay, whereas (-)-epicatechin showed very little activity (1.9 μmol of QE/100 μmol) [32]. Furthermore, the 4-methylcatechol contents were effectively the same in these two solvent extracts. Therefore, the main cellular antioxidant activity of the litchi pulp was attributed to 4-methylcatechol. The cellular antioxidant activities of common fruits have been reported as follows: wildberry ranked first (292 μmol of QE/100 g FW), followed sequentially by blackberry (232 μmol of QE/100 g FW), raspberry (114 μmol of QE/100 g FW), cranberry (47.9 μmol of QE/100 g FW), plum (33.5 μmol of QE/100 g FW), cherry (27.4 μmol of QE/100 g FW), apple (21.9 μmol of QE/100 g FW), red grape (16.3 μmol of QE/100 g FW), kiwifruit (16.1 μmol of QE/100 g FW), mango (15.3 μmol of QE/100 g FW) and pineapple (14.8 μmol of QE/100 g FW) [24]. The litchi cultivar *Feizixiao,* which is the most popular litchi cultivar in southern China, exhibited better cellular antioxidant activity than the aforementioned fruits except wildberry, blackberry and raspberry.

The ORAC and CAA values of the bound phenolic compounds from the litchi pulp residue obtained by acid hydrolysis were higher than those obtained by alkaline hydrolysis, because the contents of the bound phenolic compounds were higher in the former case. Our previous study found that the FRAP and DPPH scavenging capacities of the bound phenolic compounds from litchi pulp were positively correlated with the phenolic and flavonoid contents [19]. It can be concluded that the litchi cultivar *Feizixiao* represents a good dietary antioxidant supplement fruit.

## Conclusions

The free and bound phenolic compound contents, profiles and antioxidant activities of the extracts of litchi pulp were investigated and found to be significantly affected by the solvent system used for the extraction process. Of the five solvent mixtures evaluated in the current study for the extraction of the free phenolic compounds from litchi pulp, the use of an aqueous acetone mixture yielded the highest total contents of phenolic, flavonoid and tannin compounds, and exhibited the highest antioxidant activity. The use of an acid hydrolysis method resulted in higher extraction efficiency and antioxidant activity for the bound phenolic compounds in litchi pulp than an alkaline method. Nine individual phenolic compounds were detected in the aqueous acetone extract, and seven individual phenolic compounds were released by acid hydrolysis. The contents of both the free and bound individual phenolic compounds were affected by the choice of extraction solvent. Litchi pulp is rich in phenolic compounds and has good cellular antioxidant capacity.

## Competing interests

The authors have declared that they have no competing interests.

## Authors’ contributions

MWZ conceived of this study and designed the experiments. DXS, FLH and JXG performed the experiments. All of the authors including MWZ analysed the data and discussed the results. RFZ and DXS drafted the manuscript with the help of MWZ. All of the authors have read and approved the final manuscript.

## Pre-publication history

The pre-publication history for this paper can be accessed here:

http://www.biomedcentral.com/1472-6882/14/9/prepub
